# Supplementary material for: Improved Cladocopium goreaui Genome Assembly Reveals Features of a Facultative Coral Symbiont and the Complex Evolutionary History of Dinoflagellate Genes
Source: Microorganisms. 2022 Aug 17;10(8):1662. doi: 10.3390/microorganisms10081662 (PMC9412976; doi:10.3390/microorganisms10081662)
Supplement: Supplementary file 1 [file microorganisms-10-01662-s001.zip › Chen_SuppFigures_R1_v1.1.pdf]

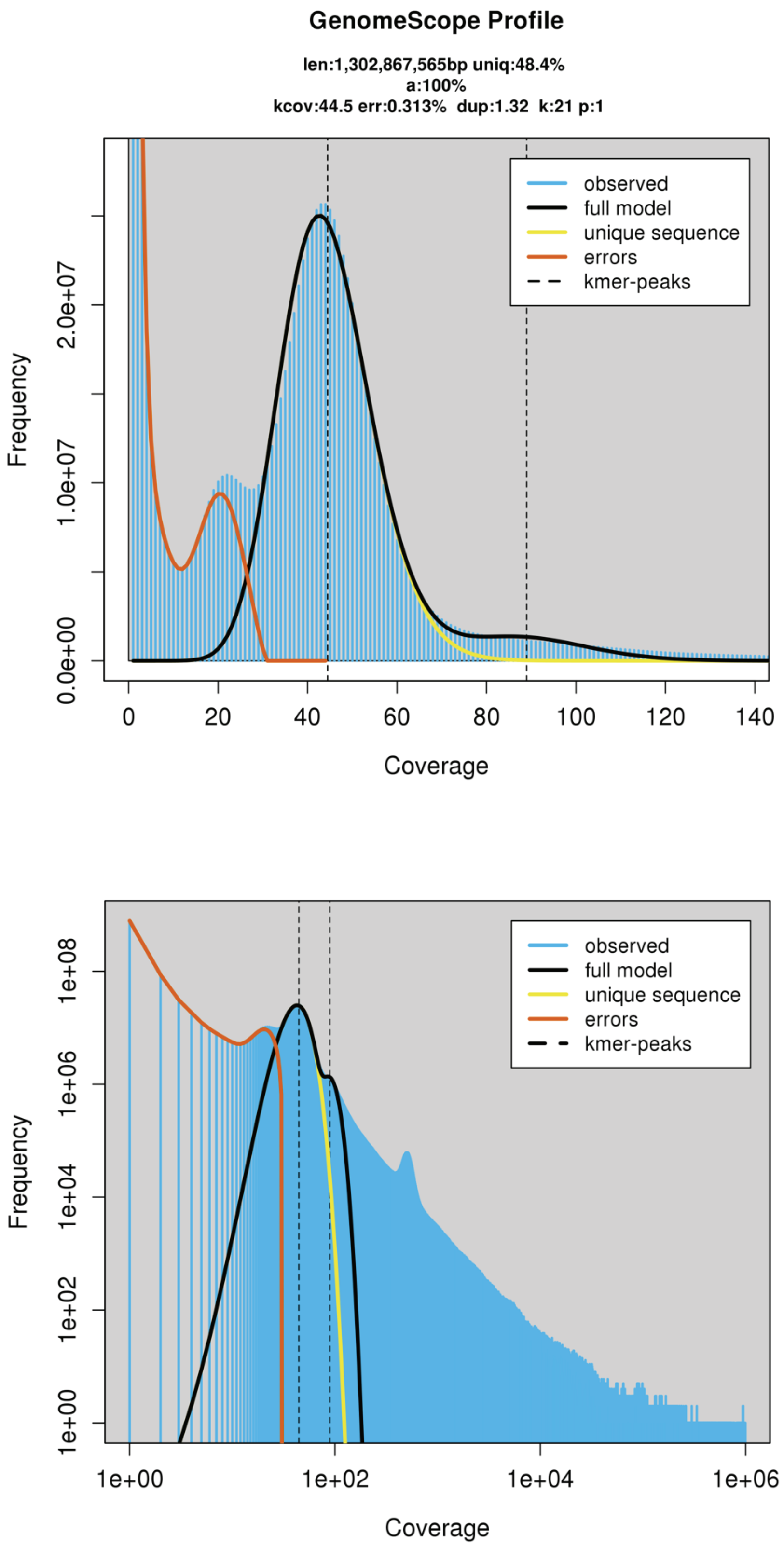

Figure S1. Genome size estimation for Cladocopium goreaui using GenomeScope v2.0, based on frequency distribution of k-mers from short-read sequence data, shown for exact (top) and log-transformed (below) values**.**

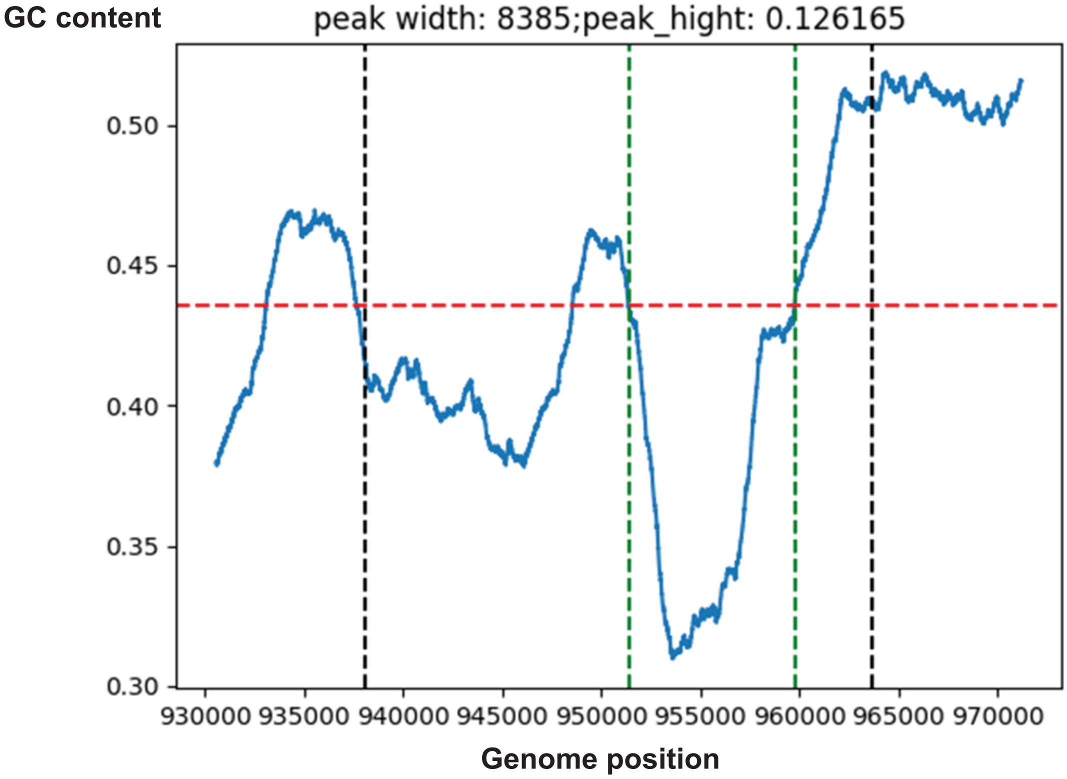

Figure S2. An example of G+C dip observed in the C. goreaui genome of a putative boundary of topologically associated domain (TAD), shown for 4000-bp sliding windows on scaffold scf7180000355754. The x-axis shows the centre position of each sliding window along the scaffold. Of the dashed lines, the red line indicates the mean %G+C of the scaffold (as back-ground), the green lines signify the G+C dip region, and the black lines signify a putative TAD boundary.

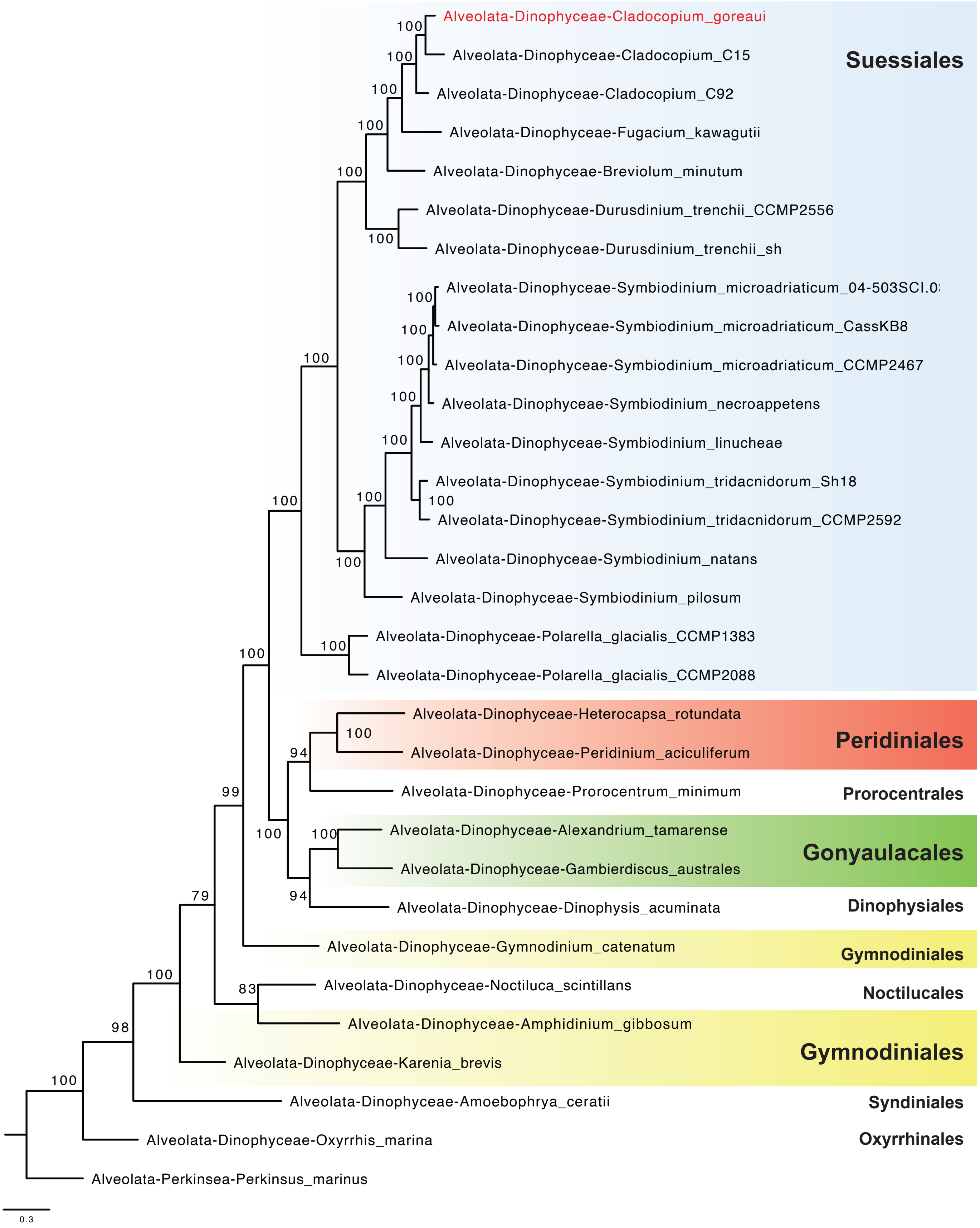

Figure S3. Species tree of 30 dinoflagellate taxa and *Perkinsus* as outgroup, inferred from 3,411 strictly orthologous (single-copy) protein sets.

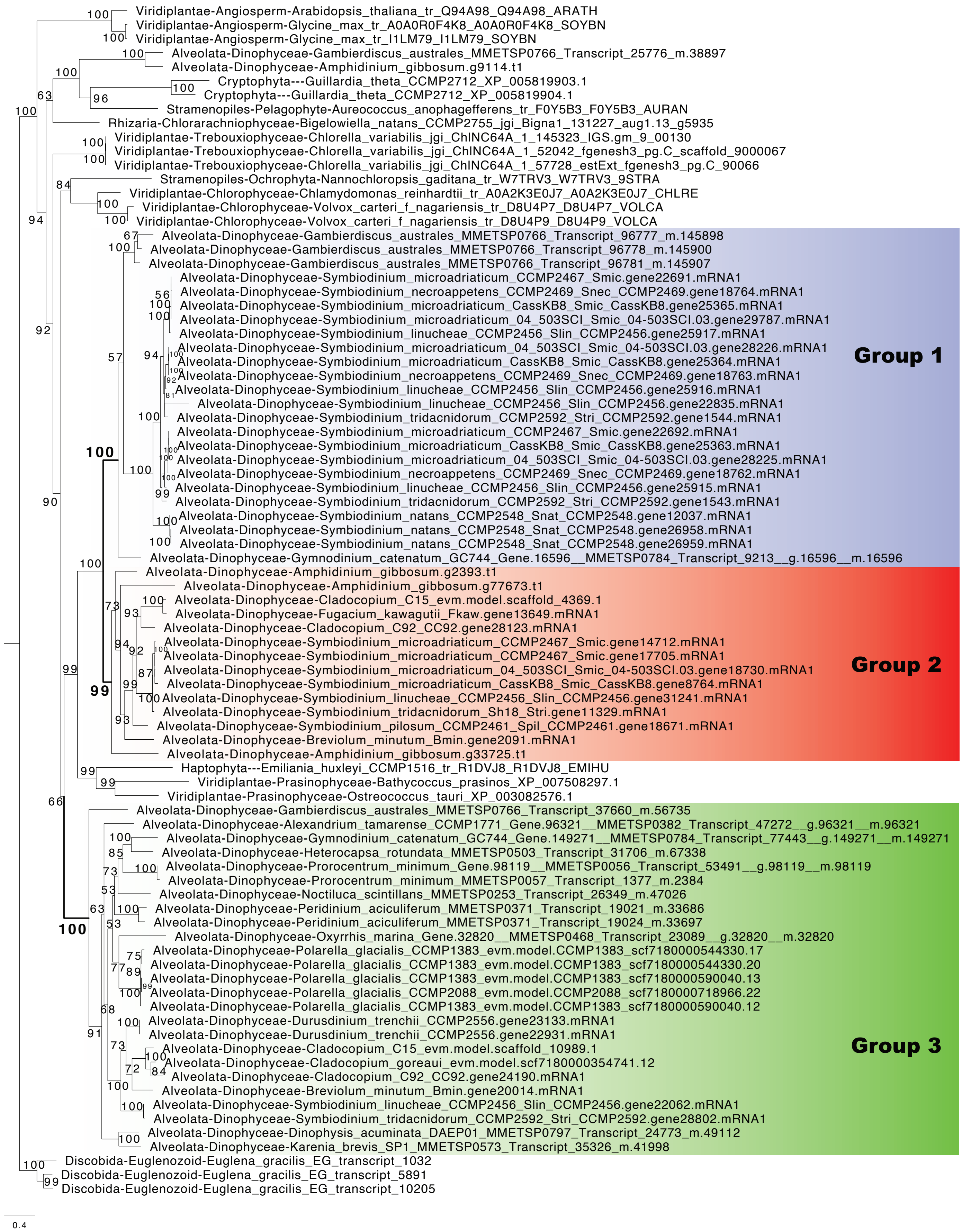

Figure S4. Maximum likelihood tree showing gene expansion of a green algal derived protein family that contain a remote homolog in Arabidopsis thaliana with function implicated in cytokinesis and meiosis.

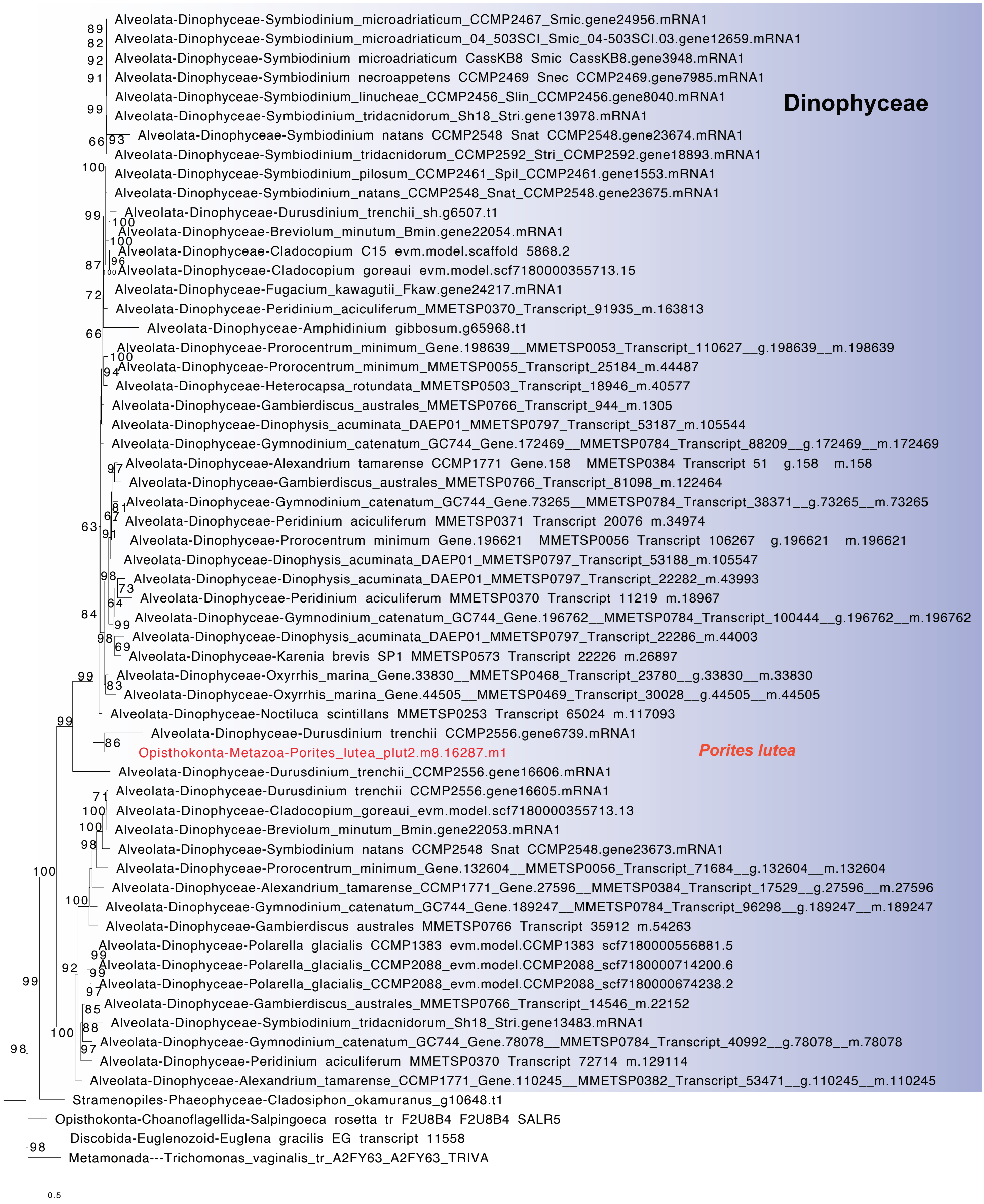

Figure S5. Maximum likelihood tree of phosphatidylinositol 4-phosphate 5-kinase showing possible misidentification of the sequence from the dinoflagellate symbiont associated with the coral.

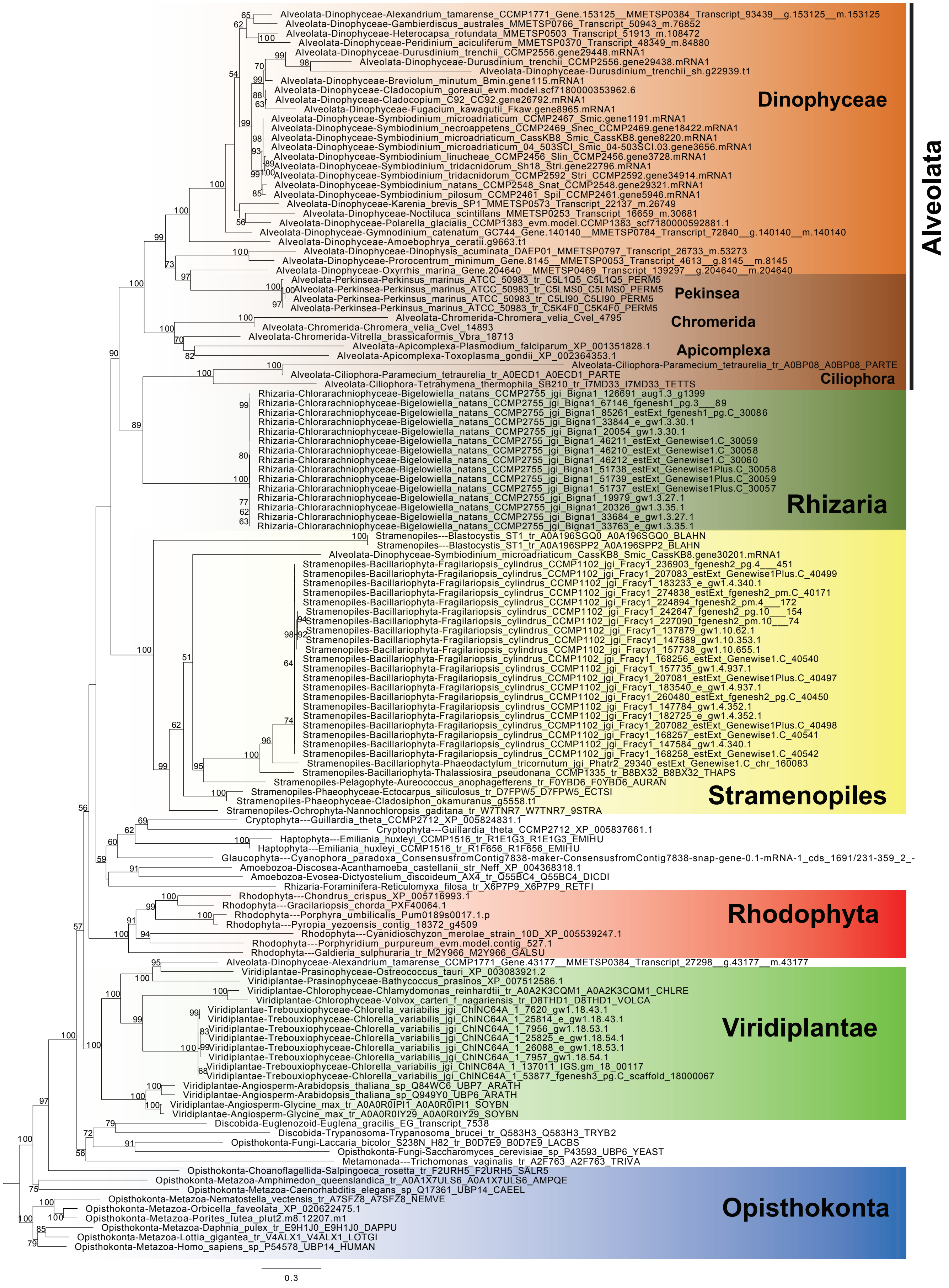

Figure S6. Maximum likelihood tree of ubiquitin carboxyl-terminal hydrolase showing strong evidence of vertical inheritance

Alveolata-Dinophyceae-Cladocopium_goreaui_evm.model.scf7180000355862.5
(autophagy-related protein 18a)

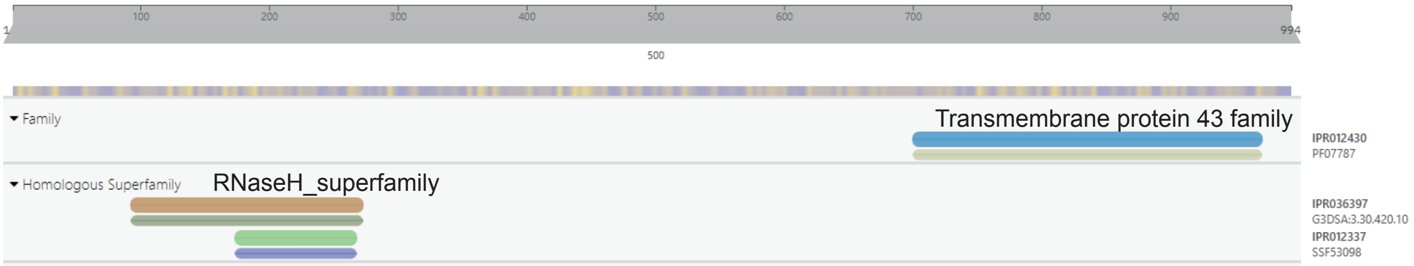

Alveolata-Dinophyceae-Cladocopium_goreaui_evm.model.scf7180000353294.2
(transmembrane protein 43)

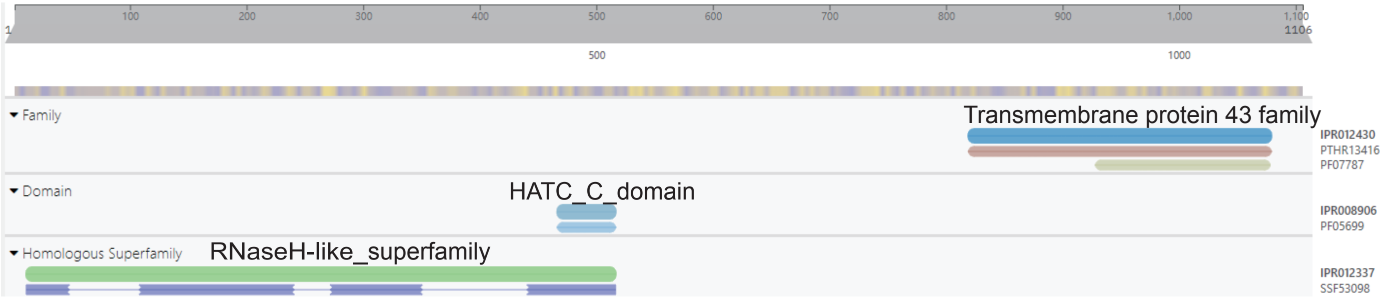

Alveolata-Dinophyceae-Symbiodinium_microadriaticum_CCMP2467_Smic.gene13716.mRNA1
(pentatricopeptide repeat-containing protein GUN1)

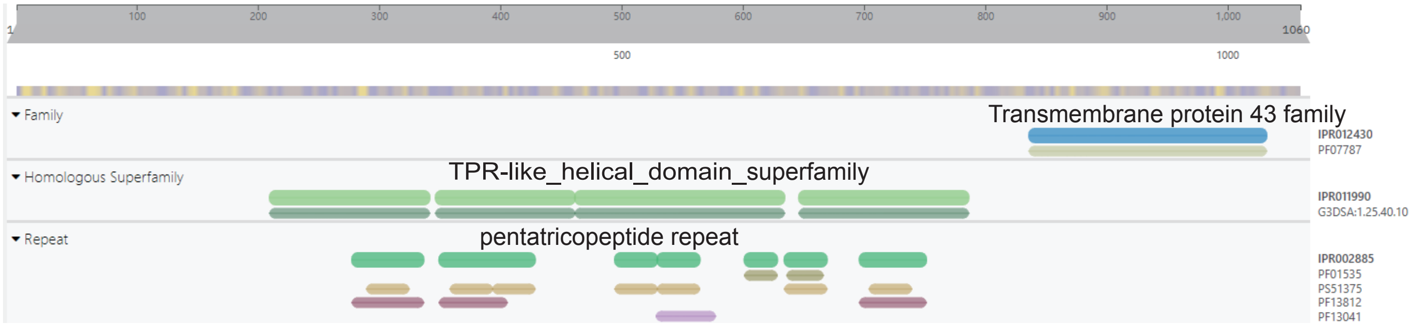

Figure S7: Domain configuration for a representative sequence from each of the three sub-clades in the tree of Figure 5, shown for the autophagy-related protein 18a, the transmembrane protein 43, and the pentatricopeptide repeat-containing protein GUN1.

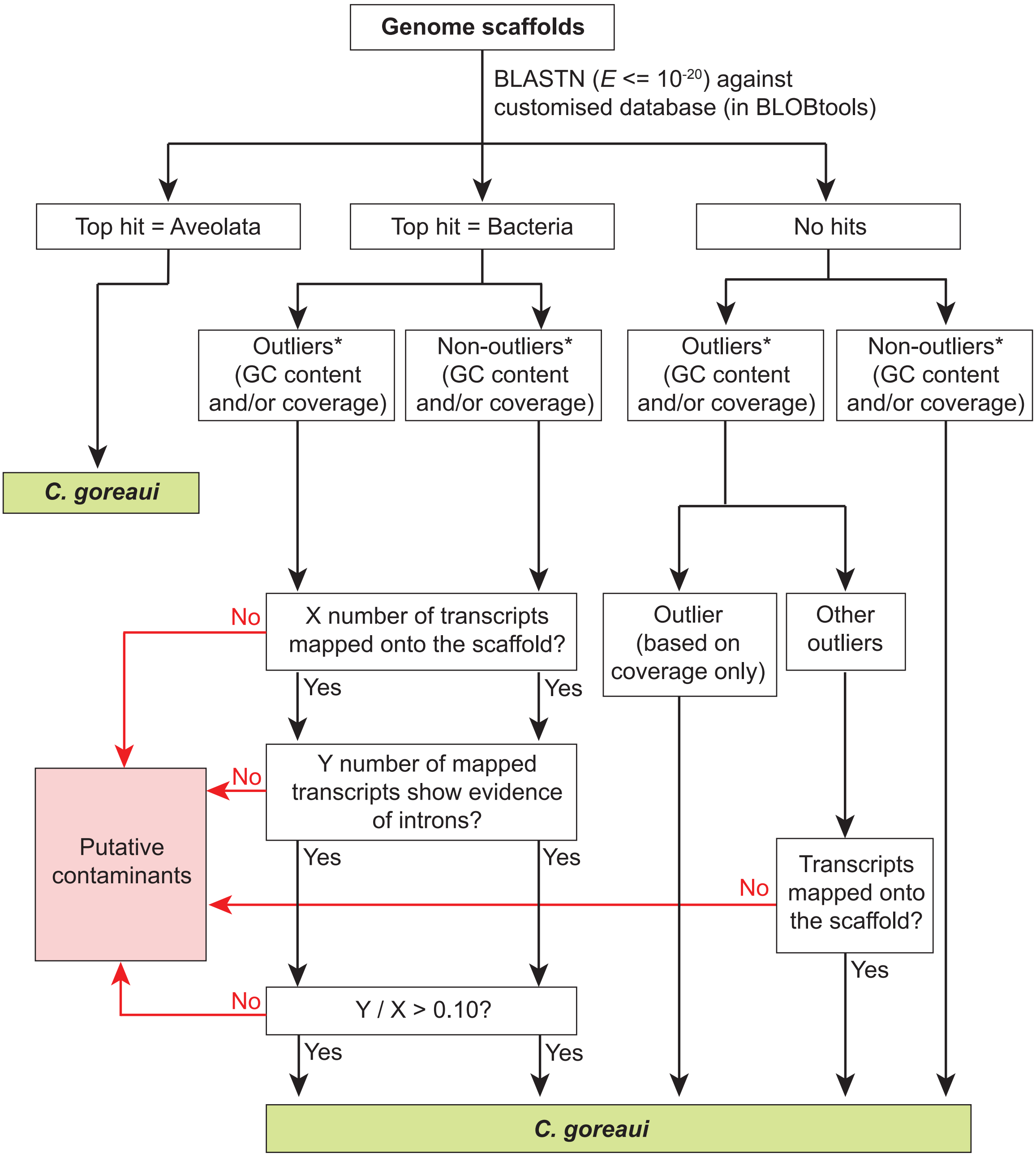

*: outliers are determined using BLOBtools. Scaffolds for which G+C content and/or read coverage is external to the range of median ± 1.5 × interquartile range (IQR) are considered as outliers.

Figure S8. Decision tree for identification and removal of putative contaminant sequences.
